# Taming the Autophagy as a Strategy for Treating COVID-19

**DOI:** 10.3390/cells9122679

**Published:** 2020-12-13

**Authors:** Blanca Estela García-Pérez, Juan Antonio González-Rojas, Ma Isabel Salazar, Carlos Torres-Torres, Nayeli Shantal Castrejón-Jiménez

**Affiliations:** 1 Department of Microbiology, Escuela Nacional de Ciencias Biológicas, Instituto Politécnico Nacional, Prolongación de Carpio y Plan de Ayala S/N, Col. Santo Tomás, Alcaldía Miguel Hidalgo, Mexico City 11340, Mexico; jgonzalezr1811@alumno.ipn.mx (J.A.G.-R.), misalazar@ipn.mx (M.I.S.); 2 Sección de Estudios de Posgrado e Investigación, Escuela Superior de Ingeniería Mecánica y Eléctrica, Unidad Zacatenco, Instituto Politécnico Nacional, Gustavo A. Madero, Mexico City 07738, Mexico; ctorrest@ipn.mx; 3 Área Académica de Medicina Veterinaria y Zootecnia, Instituto de Ciencias Agropecuarias-Universidad Autónoma del Estado de Hidalgo, Av. Universidad km. 1. Exhacienda de Aquetzalpa A.P. 32, Tulancingo, Hidalgo 43600, Mexico; nayeli_castrejon@uaeh.edu.mx

**Keywords:** SARS-CoV-2, COVID-19, autophagy, inflammation, cytokine storm, obesity

## Abstract

Currently, an efficient treatment for COVID-19 is still unavailable, and people are continuing to die from complications associated with SARS-CoV-2 infection. Thus, the development of new therapeutic approaches is urgently needed, and one alternative is to target the mechanisms of autophagy. Due to its multifaceted role in physiological processes, many questions remain unanswered about the possible advantages of inhibiting or activating autophagy. Based on a search of the literature in this field, a novel analysis has been made to highlight the relation between the mechanisms of autophagy in antiviral and inflammatory activity in contrast with those of the pathogenesis of COVID-19. The present analysis reveals a remarkable coincidence between the uncontrolled inflammation triggered by SARS-CoV-2 and autophagy defects. Particularly, there is conclusive evidence about the substantial contribution of two concomitant factors to the development of severe COVID-19: a delayed or absent type I and III interferon (IFN-I and IFN-III) response together with robust cytokine and chemokine production. In addition, a negative interplay exists between autophagy and an IFN-I response. According to previous studies, the clinical decision to inhibit or activate autophagy should depend on the underlying context of the pathological timeline of COVID-19. Several treatment options are herein discussed as a guide for future research on this topic.

## 1. Introduction

The pandemic of coronavirus disease 2019 (COVID-19) caused by the severe acute respiratory syndrome coronavirus 2 (SARS-CoV-2) represents an enormous challenge for the scientific community around the world. There are numerous reports on the origin of the new virus as well as the diagnosis, prevention, and treatment of its associated disease. The suggested therapeutic regimens have been subjected to clinical studies, finding positive outcomes in some cases and a lack of significance (compared to standard care) in others (Table 1). Since the drugs at this time administered to treat COVID-19 patients do not have significant efficacy to counteract the effects of the disease, new therapeutic options are urgently needed to control the infection to avoid fatal outcomes in hospitalized patients that have developed a severe infection.

The pathogenesis of COVID-19 is complex and poorly understood. It is manifested as a wide spectrum of symptoms and complications, including pneumonia, acute respiratory distress syndrome (ARDS) and endothelial dysfunction [18,22,23]. The condition of symptomatic infected individuals ranges from mild to severe. One of the components linked to severe cases of COVID-19 is an excessive host immune response characterized by uncontrolled inflammation derived from cytokine storm syndrome [18,24,25]. Of considerable concern is the relation between the development of severe COVID-19 and comorbidities among the elderly, especially obesity, hypertension, and diabetes.

According to research on the mechanisms of COVID-19, one of the fundamental cell processes in the pathogenicity of the disease could possibly be autophagy. This sophisticated, highly orchestrated process represents an essential activity of cell remodeling and maintenance. Autophagy is responsible for selectively eliminating dysfunctional organelles, intracellular pathogens and misfolded proteins as well as regulating the immune response [26,27,28,29]. On the other hand, defective autophagy is associated with obesity, cancer, neurodegenerative disorders and autoimmune and infectious diseases [30,31,32,33,34]. Additionally, autophagic activity declines in the elderly [35]. However, no consensus exists on the usefulness of inhibiting or stimulating this mechanism to restrict the replication of SARS-CoV-2 to limit the clinical complications of COVID-19. The main reason for the hesitation to intervene in the process of autophagy is the complex relationship that it holds with multiple cell phenomena contributing to the maintenance of homeostasis. In the current review, the antiviral and anti-inflammatory roles of autophagy are emphasized as a plausible therapeutic approach for re-establishing homeostasis in patients suffering from COVID-19.

## 2. Brief Overview of the New SARS-CoV-2

SARS-CoV-2 belongs to the family *Coronaviridae* and order *Nidovirales*. It corresponds to a group of enveloped viruses with positive sense single-stranded ribonucleic acid (ssRNA) genomes able to affect numerous species like birds or terrestrial and aquatic mammals (including humans). Of the six coronaviruses known to infect humans, highly pathogenic SARS-CoV and Middle East respiratory syndrome coronavirus (MERS-CoV) cause acute respiratory syndromes [36,37]. The phylogenetic analysis shows a higher similarity of the new SARS-CoV-2 to SARS-CoV than MERS-CoV [38]. The origin of SARS-CoV-2 is being widely discussed and theoretical scenarios have been published [39].

The details of the unique features of SARS-CoV-2 that allow it to enter human cells and promote a replication process are not completely understood. Spike (S) type I glycoprotein, one of the four structural proteins in SARS-CoV-2, interacts with angiotensin-converting enzyme 2 (ACE2) on the cell surface [40,41]. ACE2, found in epithelial cells of lung, kidney, intestinal and other tissues, is overexpressed during the development of some chronic diseases such as hypertension and diabetes mellitus. The overexpression of ACE2 increases the risks involved in COVID-19 [41,42,43].

The coronavirus S glycoprotein is composed by two functional subunits, S1 and S2. S1 is responsible for the binding of the virus to ACE2 receptors and S2 for the fusion of viral and host cell membranes. The SARS-CoV-2 S glycoprotein harbors furin and TMPRSS2 cleavage sites, both essential for the proteolytic activation of the S protein and the expansion of the virus. Hence, it is speculated that the virus has a high level of tropism and pathogenicity [44,45].

Replication of a coronavirus starts when the receptor-binding motif (RBM) in the receptor-binding domain (RBD) of the S glycoprotein is identified by human ACE2 [46]. Afterwards, the cell uptakes the viral particle, which remains concealed inside an endosome. These conditions allow the release of genomic RNA into the cytoplasm of the host cell, where the virus exploits the entire cell machinery, not only to replicate its RNA but also to synthesize viral proteins and assemble new viral particles. Several mechanisms and molecules (both from the virus and the host) participate in the binding of coronavirus to host cells and its subsequent replication once inside the cells. It is necessary to better grasp the interplay of these phenomena in order to design drugs to counteract viral infection and its effects.

The formation of double-membrane vesicles (DMVs) as sites of viral RNA replication and transcription has been observed in some coronaviruses. Therefore, autophagy or its components are key elements in viral infection [47,48], and they should be considered important as a target to avoid SARS-CoV-2 replication.

## 3. The Autophagy-Coronavirus Relationship

In recent years, much research has been focused on xenophagy, the process by which a cell directs autophagy to sense and control the growth of intracellular microorganisms (e.g., bacteria, parasites, and viruses) to maintain or restore homeostasis [26,30,49]. The antiviral activity mediated by autophagy encompasses two mechanisms. Firstly, it clears the virus by directly degrading viral particles (xenophagy) or destroying neosynthesized viral components (virophagy) [50]. Secondly, it induces the innate and adaptive immune systems to produce antiviral humoral and/or cell mediators.

Whereas the mechanisms of autophagy can lead to viral clearance, in other cases, they are subverted by strategies that viruses have evolved to inhibit, escape or manipulate the respective host components. Hence, viruses could possibly trigger autophagy for their own advantage at the distinct steps, during viral attachment and entry, membrane fusion, release of viral components in the host cell cytoplasm and replication [51]. During the first stage of viral infection, for example, the virus hijacks the autophagosomes and utilizes them as replicative niches or to modulate autophagy as a means to downregulate the antiviral mechanisms of cells.

Since autophagy is likely to be the proverbial double-edged sword [30], the clinical application of its activators or inhibitors must await a more in-depth understanding of the interplay between the corresponding viral and host components. To explore this interplay, it is useful to begin with reports on the role of autophagy in other coronavirus models.

### 3.1. Direct Coronavirus-Autophagy Interplay

The first finding about the relation between autophagy and coronavirus replication was the discovery of the formation of DMVs resembling autophagosomes as sites for viral RNA replication and transcription. It has been suggested that the formation of DMVs during the infection process of mouse hepatitis virus (MHV) is associated with the LC3 protein [52], a key marker of autophagosomes. However, neither ATG5 nor an intact autophagic pathway are required for MHV replication or release [53]. In contrast, Gammacoronavirus infectious bronchitis virus (IBV) induces the formation of autophagosomes and autophagic flux via the mitogen-activated protein kinase extracellular signal-regulated kinase 1/2 (MAPK/ERK1/2) pathway, dependent on ATG5 but independent of myosin-like BCL2 interacting protein (Beclin1). The latter is one of the most important autophagy master regulators [54]. Similarly, transmissible gastroenteritis virus (TGEV) triggers complete autophagic flux and the autophagy elicited by rapamycin inhibits its replication [55]. Moreover, porcine epidemic diarrhea virus (PEDV) infection enhances autophagy flux in the Vero cell line model and its pharmacological inhibition by 3-methyladenine (3-MA) significatively decreases the viral titers [56]. Contrarily, in porcine intestinal epithelial cells, PEDV infection is suppressed by the autophagic flux triggered by rapamycin [57]. A screening of non-structural proteins (NSPs) of IBV, MHV and SARS-CoV showed that the inhibition of autophagy dependent on mTOR can be induced by NSP6 [58]. In the endoplasmic reticulum, NSP6 of IBV gives rise to the formation of autophagosomes with a small diameter and therefore may limit autophagosome expansion and compromise the formation of autolysosomes [59]. 

According to recent studies, some coronavirus-related proteins hijack critical regulators of autophagy to modulate this host process to the advantage of the virus, allowing its successful replication. The NSPs of human coronavirus (hCoV) (e.g., membrane-associated papain-like protease 2 and PLP2-TM) and their homologues (e.g., SARS-CoV PLpro-TM, PEDV PLP2-TM and MERS-CoV PLpro-TM) induce incomplete autophagy and promote the capacity of Beclin1 to hijack the stimulator of interferon genes (STING), which is a crucial regulator of antiviral type I interferon (IFNs-I) signaling. The result is the disabling of the antiviral response and the potential of the virus to efficiently replicate in HEK293T cells [60]. MERS-CoV replication in Vero cells causes decreased expression of Beclin1 stemming from increased phosphorylation of S-phase kinase-associated protein 2 (SKP2) at S72, leading to a decline in the formation of autolysosomes. Importantly, the inhibition of SKP2 enhances autophagy and consequently reduces the replication of MERS-CoV in this model [61].

**Figure 1:**
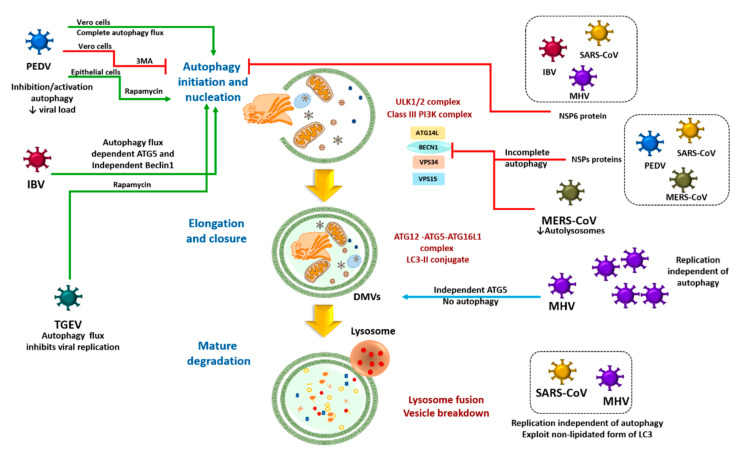
Schematic coronaviruses-autophagy relationship. The figure shows the main findings related to the inhibition or triggering of autophagy by coronaviruses. Green lines represent the complete activation of autophagy by coronaviruses. Red lines show inhibition of autophagy or incomplete autophagy. Blue line indicates the double-membrane vesicles (DMVs) formation by coronaviruses independent of autophagy. In some cellular models, SARS-CoV and mouse hepatitis virus (MHV) exploit the non-lipidated form of LC3 and its replication is independent of autophagy.

On the other hand, various authors have proposed that the formation of DMVs as replication niches of equine arteritis virus (EAV), MHV and SARS-CoV occurs independently of autophagy because the virus takes advantage of the non-lipidated form of LC3 (Figure 1) [62,63]. Thus, the latter molecule has an additional role (independent of autophagy) as part of the membrane-bound receptor, which is to guarantee endoplasmic-reticulum-associated protein degradation (ERAD). This makes it a possible target for the inhibition of viral replication [64]. Since the replication of SARS-CoV can be affected by the use of pharmacological inhibitors of m-calpain [65], a cysteine protease involved in the regulation of the cytoskeleton [66], such replication apparently does not depend on the functional ubiquitin-proteasome system (UPS) or autophagy pathway. Due to the scant information available and the diversity of experimental models employed, there is no clear view of how autophagy operates in relation to the clearance of coronaviruses. Is it a mechanism to prevent or promote viral replication? 

The sterilizing effect of autophagy was first beautifully demonstrated in *Mycobacterium tuberculosis* infections, in which the stimulation of autophagy suppressed the intracellular survival of the bacterium [67]. Nevertheless, the result of fomenting autophagy remains unclear in viral infections and requires further investigation. The pharmacological triggering of autophagy by the intake of vitamin D and histone deacetylase inhibitors (HDACi) was recently reported to limit HIV replication in macrophages [68,69]. Contrarily, the activation of autophagy in CD4+ T cells has been a stimulus for HIV replication [70,71]. Kurarinone, a prenylated flavanone, inhibits HCoV-OC43 infection in human lung MRC-5 cells by impairing virus-induced autophagy [72]. Hence, more research is needed to understand the conditions allowing the pharmacological stimulation or inhibition of autophagy to impede the replication of coronaviruses.

### 3.2. Indirect Coronavirus–Autophagy Interplay

In addition to the direct elimination of viruses, autophagy participates in the regulation of the antiviral immune response. The signaling for the generation of IFNs-I, the key immune response against viruses, is regulated by autophagy at different levels. Autophagy along with the UPS are responsible for ensuring the delivery of viral cytosolic pathogen-associated molecular patterns (PAMPs) to pattern recognition receptors (PRRs), triggering downstream signaling to produce an IFN-I response [73]. On the other hand, through the degradation of signaling molecules, autophagy negatively regulates the IFN-I signaling pathway to avoid an excessive and persistent immune response [74]. The available evidence about the interplay between autophagy and IFN-I responses during a coronavirus infection is inconclusive. Nonetheless, coronaviruses are known to use various mechanisms to overcome the IFN-I response. Thus, a role of autophagy for the same purpose would not be surprising.

It is necessary to consider the function of IFN-I in the host immune response before returning to the discussion of its possible regulation by autophagy. Upon recognition of viral PAMPs by host cell PRRs, the IFN-I response is activated. The IFN-I response directly inhibits viral replication and indirectly modulates the immune response to viral infection [75]. The major antiviral PRRs are divided into three groups of receptors located in the endosomal environment: TLRs, RLRs and NLRs (Toll-like, RIG-1-like, and NOD-like receptors, respectively) [76,77,78,79,80,81,82]. The engagement of PRRs triggers multiple signaling cascades that converge in the generation of IFN-Is and other cytokines. Subsequently, IFN molecules bind to their cell surface receptors to amplify the antiviral response by stimulating the downstream Janus kinase signal transducer and activator of transcription (JAK-STAT) pathway, prompting the transcription of hundreds of IFN-regulated genes (IRGs) with potent antiviral activity [83].

Research about the participation of IFN-I in the immune response to an infection by SARS-CoV-2, SARS-CoV and MERS-CoV is in progress. SARS-CoV and MERS-CoV delay IRG expression until after they reach peak viral titers [75,84]. Similarly, SARS-CoV-2 does not elicit a robust IFN-I response, even during viral replication [85,86,87]. All three coronaviruses are reported to interfere with the generation of an IFN-I response, indicating the evolution of strategies of immune response evasion.

The downregulation of IFN-I may be controlled at a transcriptional or translational level. For instance, MERS-CoV-induced modifications in histones downregulate the expression of subsets of IFN-stimulated genes (ISGs) [84]. Moreover, the MERS-CoV nucleocapsid protein antagonizes the production of both type I and type III IFNs by scavenging TRIM25, an E3 ubiquitin ligase essential for triggering the RIG-I signaling pathway [88]. SARS-CoV and SARS-CoV-2 interfere with the IFN signaling pathway through their viral proteins, such as NSPs and open reading frames (ORFs). The latter viral proteins antagonize the generation of INF-Is or inhibit host mRNA translation by interacting with the molecules implicated in the pathways of the RIG-I and MDA5 signaling sensors [89]. It is possible that these coronaviruses downregulate the JAK-STAT pathway to inhibit the IFN-I response [90].

Some viral models have provided vital information on how autophagy operates to inhibit the IFN-I defenses of cells. The first studies revealed the negative interplay between autophagy and the IFN-I response, and recent research has explained the mechanisms involved. In Japanese encephalitis virus (JEV) infection, for instance, the Atg5 and Atg7 autophagy proteins are negatively correlated with the activation of interferon regulatory factor 3 (IRF3), a marker of the IFN-I signaling pathway triggered by the virus [91]. In the hepatitis C virus (HCV) model, autophagy chemically enhanced by the unfolded protein response (UPR) inhibits IFN-β activation [92]. Similarly, vesicular stomatitis virus (VSV) infection stimulates autophagy through a process mediated by Atg5, and the Atg5-Atg12 conjugate and NLRX1–TUFM complex negatively regulate the IFN-I pathway by direct association with the retinoic acid-inducible gene I (RIG-I) and IFN-β promoter stimulator 1 (IPS-1) through the caspase recruitment domains (CARDs) [93,94].

**Figure 2:**
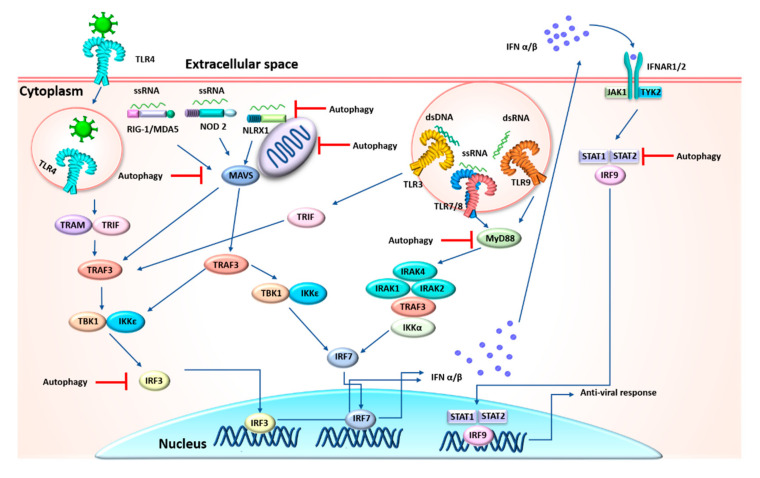
Negative regulation of interferons (IFNs) by autophagy. Upon recognition of viral pathogen-associated molecular patterns (PAMPs) by pattern recognition receptors (PRRs), several signaling pathways are activated to produce IFNs. This response is amplified by the activation of the Janus kinase signal transducer and activator of transcription (JAK-STAT) pathway. Some coronaviruses negatively regulate the IFNs’ response via the autophagic degradation of key molecules downstream of IFN signaling cascade (e.g., IRF3, MAVS, NLRX1, mitochondria, MyD88 and STAT).

Interestingly, the regulation of the IFN-I response by autophagy seems dependent on the pathways forming part of mitochondrial functionality. Atg5−/− cells increase the levels of IPS-1 and mitochondrial reactive oxygen species (ROS), which in turn amplifies RLR signaling [95]. SARS-CoV impairs the IFN response by using the open reading frame-9b (ORF-9b) to target altered mitochondria and the MAVS/TRAF3/TRAF6 signalosome (TRAFs are TNF-associated factors) [96]. One study conclusively demonstrated that the suppression of IFN-β in cells infected by transmissible gastroenteritis coronavirus was dependent on mitophagy stimulated by doxycycline [97]. Moreover, the autophagic degradation of STAT2 gives rise to the blockage of IFN signaling [98]. Autophagy is also implicated in the suppression of the TLR7-mediated IFN-I signaling pathway [99]. According to the aforementioned reports, autophagy is one of the host cell processes exploited by coronaviruses to negatively regulate the IFN-I pathway (Figure 2).

The literature provides no clear evidence of viral clearance directly carried out by autophagy. However, there are numerous descriptions of the blockage of the antiviral immune response with the participation of autophagic components. In the first pathological stage of COVID-19, SARS-CoV-2 is recognized by ACE2 receptors, followed by its propagation and migration down the respiratory tract. The subsequent clinical manifestations [100] are mainly due to the viral infection itself [101]. In order to restrict viral replication in this phase, the immune response must be well-controlled and orchestrated by means of the activation of cells of the innate defense. Otherwise, the immune response can be manipulated by the virus to damage the organism and allow for replication [102]. Therefore, the inhibition of autophagy could possibly be an alternative strategy to restore the antiviral effectiveness of the immune response. Conclusive studies are still lacking, but potent in vitro inhibition of SARS-CoV-2 replication was recently reported for VPS34-IN1 and VVPS34-IN1 and their analogues, which are inhibitors of vacuolar protein sorting (VPS) 34 (a class III phosphoinositol-3 kinase, PI3K). These inhibitors block autophagy [103,104].

## 4. Anti-Inflammatory Function of Autophagy

The hallmark of COVID-19 is the diversity of symptoms and complications that lead to a fatal outcome. Whereas most individuals are thought to present mild to moderate symptoms upon infection with SARS-CoV-2, approximately 12–15% develop serious complications such as pneumonia and acute respiratory distress syndrome (ARDS) [18,22] associated with late-stage critical care [100]. COVID-19-induced ARDS is characterized by difficulty in breathing and decreased blood oxygen levels [24]; these circumstances eventually result in systemic respiratory failure and death. There is conclusive evidence that a delayed or absent IFN-I and IFN-III response together with robust cytokine and chemokine production are major factors in the development of severe COVID-19 [85,102,105], as this combination of conditions implies uncontrolled inflammation and cytokine storm syndrome [18,24,25].

Elevated levels of the chemokine MCP1 and the various cytokines (e.g., IL-6, IL-1β, TNF-⍺, IL-10 and IL-8) have been well documented in patients with severe COVID-19. Cytokine release syndrome is usually initiated by macrophages, dendritic cells, NK cells and T cells in response to PRR identification of PAMPs [106]. In COVID-19, the deregulated activation of macrophages has a substantial role in increasing the level of cytokines [107]. The participation of epithelial and endothelial cells in the inflammatory response to COVID-19 is still unclear. These cells produce a large quantity of CCL3, CCL5, CCL2 and CXCL10 during a SARS-CoV infection, while they generate significant but delayed IFN and pro-inflammatory cytokine (IL-1β, IL-6 and IL-8) responses during a MERS-CoV infection [108,109]. Hence, inflammation fostered by COVID-19 may affect the vascular system and also it can contribute to microcirculatory lesions. Acceleration of vascular inflammation can be derived from an imbalance in cytokine production, the activation of macrophages associated with the release of pro-coagulant factors, such as plasminogen activators, and the enhanced expression of PAI-I. Additionally, it promotes a prothrombotic state in patients with a severe stage of the disease, resulting in higher levels of IL-6 and D-dimer [110].

A viral infection engages PRRs by means of PAMPs and damage-associated molecular patterns (DAMPs), thus triggering a cascade along several signaling pathways that causes an inflammatory response. The best-characterized viral sensors are TLRs, RLRs and NLRs [111,112,113]. In TLR signaling pathways, there are two key adaptors: the myeloid differentiation factor 88 (MyD88) and the Toll interleukin (IL)-1 receptor homologue, being the Toll/Interleukin-1R (TIR)-domain-containing adaptor-inducing INF-β (TRIF) [114,115]. These adaptors elicit an antiviral response by stimulating signaling proteins downstream, including the transcriptional nuclear factor κB (NF-κB) and IRF3 or cascades of MAPKs, which give rise to the expression of genes encoding for inflammatory cytokines (e.g., IL-6, IL-1, IL-12, TNF-⍺ and IFN-Is) [116].

Upon activation of RLRs, RIG-I and/or MDA5 translocate to the mitochondria, where they interact with mitochondrial antiviral-signaling (MAVS) proteins (IPS-1, VISA and Cardif) [117,118,119] and, through CARDs, interact with several protein kinases (e.g., TBK1 and IKKἐ). These protein kinases phosphorylate and activate transcription factors (IRF and NF-κB) to induce the transcription of a variety of innate immune response genes involved in antiviral activity, such as genes encoding IFNs as well as those involved in an antiviral and pro-inflammatory response.

The triggering of NLRs leads to the assembly of inflammasomes, which are large protein complexes [120] that foment inflammation. They are characterized by the presence of a central nucleotide-binding and oligomerization (NACHT) domain flanked by C-terminal leucine-rich repeats (LRRs) and the N-terminal CARD or pyrin domain (PYD) [121]. NLRP3 is currently the most fully characterized inflammasome and consists of the NLRP3 scaffold, the ASC (PYCARD) adaptor and caspase-1. The active caspase-1 p10/p20 tetramer acts on cytokine pro-IL-1β to generate the active molecules [122] that efficiently promote an inflammatory reaction and cell death via pyroptosis [73]. The E protein from SARS-CoV and viroporin 3a was recently reported to activate the NLRP3 inflammasome [123,124].

Since the main characteristic of severe COVID-19 is a deregulated inflammatory process, among the objectives of any intervention should be regulation of the exaggerated production of immunological mediators. Over the last few years, a growing body of evidence has emphasized the importance of autophagy in regulating the innate and adaptative immune response and avoiding excessive inflammation. The strong relation between the inflammatory response and autophagy was first found in studies that linked the predisposition for chronic inflammatory disorders and autoimmune diseases with single nucleotide polymorphisms (SNPs) in genes associated with autophagy, including ATG16L1, immunity-related GTPase family M (IRGM) and LRR kinase 2 (LRRK2) [125,126,127,128,129].

Autophagy can involve several pathways to act as a negative regulator of inflammation and return to equilibrium. It removes inflammasome-activating stimuli (e.g., PAMPs and DAMPs) from the cytosol [130,131] and regulates IL-1β production by the direct destruction of inflammasomes via inflammasome ubiquitination, leading to the recruitment of p62 and LC3 [132]. Another mechanism of autophagy, denominated mitophagy (the removal of damaged mitochondria), prevents the release of cytosolic mtDNA and the accumulation of ROS. These molecules activate the NLRP3 inflammasome very effectively [133]. Damaged mitochondria are known to contribute to the promotion of the inflammatory response mediated by NLRP3 inflammasomes [134]. In some viral infections, NLRP3 is activated by mitofusins, proteins required for mitochondrial fusion [135]. The latter results have been confirmed by inhibiting mitophagy/autophagy with 3-MA and observing the accumulation of damaged mitochondria and the increased concentration of mitochondrial ROS, which elicit the secretion of IL-1β dependent on the level of NLRP3 and ASC [136]. In the macrophage model, moreover, sestrin 2 (SESN2, part of a family of stress-inducible proteins) suppresses the hyperactivation of the NLRP3 inflammasome by clearing damaged mitochondria through mitophagy, mediated by the aggregation of SQSTM1 and a boost in protein levels of ULK1 [137]. Interestingly, the NLRP3 inflammasome is also negatively regulated by NF-κB via autophagy (implying an anti-inflammatory role of this transcription factor) [138,139] and by lysosomal degradation of pro-IL-1β [140,141]. In macrophages and dendritic cells, the inhibition of autophagy leads to higher levels of both IL-1β and IL-23, and these in turn stimulate T lymphocytes to secrete IL-17, IFN-γ and IL-22 [142]. Persistent inflammatory stimuli or infection can trigger pyroptosis, which involves the abundant secretion of inflammatory cytokines. Autophagy has been reported to negatively regulate pyroptosis by eliminating the ASC pyroptosome [132,143].

The immune response generated by RLR signaling is controlled by autophagy as well. In the LRR, proteins 25 (LRRC25) and 59 (LRRC59) work together to modulate IFN-I signaling by manipulating DDX58 stability through selective autophagy [144,145]. Additionally, LRRC25 operates as an inhibitor of NF-κB signaling by promoting the interaction of p65/RelA (one of the NF-κB transcription factors) with cargo receptor p62 to facilitate its autophagic degradation [146].

**Figure 3:**
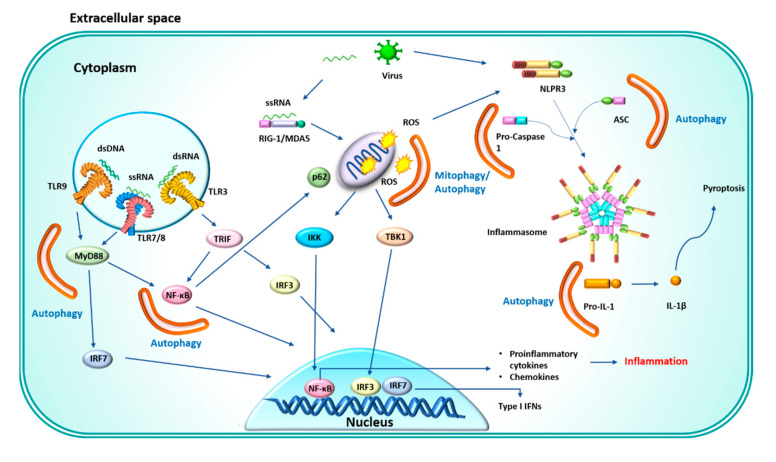
Regulation of inflammation by autophagy. The principal points downstream of proinflammatory cytokine signaling that can be potentially regulated by autophagy are indicated. The proinflammatory response might be regulated by autophagic degradation of pivotal molecules like MyD88, the nuclear factor kB (NF-κB) and depolarized mitochondria and ROS. Moreover, autophagy can regulate IL-1β production via the removal of ASC pyroptosome, pro-caspase 1 and pro-IL-1.

Pathological damage can be caused by excessive TLR signaling, which can be effectively suppressed by autophagy at multiple levels to avoid such damage. For instance, TLR signaling is intricately related to the stimulation of NLRP3 by ROS production [147], and autophagy limits excessive inflammation, maintaining the quality of mitochondria. Similarly, aggrephagy controls TLR signaling through the autophagic receptors SQSTM1, HDAC6 and NDP52. MyD88 is incorporated into sequestosomes and aggresomes by SQSTM1 and HDAC6, which suppress the TLR4-induced activation of p38 and JNK but do not clearly affect NF-κB signaling. NDP52 mediates the degradation of the TRIF–TRAF6 complex by aggrephagy, thereby suppressing TLR3/4-induced activation of NF-κB [148,149] (Figure 3). Paradoxically, autophagy has been found to be a mediator of interleukin-1β secretion in human neutrophils [150]. Overall, autophagy is a fine-tuning mechanism closely related to the inflammatory response, and its downregulation might be essential for avoiding the pathophysiology of inflammatory diseases like COVID-19.

## 5. Inflammation, Obesity and Autophagy

Unfortunately, obesity and its complications (e.g., hypertension and diabetes) are intimately related to the aggressiveness of COVID-19, and therefore they represent a risk factor for COVID-19-associated mortality [151]. This is particularly relevant for the United States and Mexico, being countries representative of a high prevalence of obesity. The first studies on patients from Wuhan, China, described a fatal outcome for 48% who had a comorbidity, such as hypertension (30%), diabetes (19%) and coronary heart disease (8%) [152]. According to a recent report, the probability of developing severe COVID-19 is 1.42-fold greater with obesity, 1.87-fold with diabetes and 1.77-fold with hypertension [153]. On 30 November (2020), the United States and Mexico ranked first and fourth, respectively, in the number of deaths caused by COVID-19. The Mexican health authorities have emphasized the great impact of obesity on the infected population with a fatal outcome.

The prevalence of obesity owes itself mainly to two factors affecting a large part of the population nowadays: a high caloric food intake and a sedentary lifestyle. Obesity is accompanied by an accumulation of dysfunctional adipose tissue, resulting in elevated levels of fatty acids, triglycerides, and low-density lipoprotein cholesterol (LDL cholesterol). This imbalance leads to an inflammatory response and a plethora of complications, including insulin resistance, hypertension, diabetes mellitus, dyslipidemia, insulin intolerance, nonalcoholic fatty liver disease, heart failure, cancer, and respiratory diseases [154]. Obesity is a multifactorial condition, consisting of several etiological mechanisms: genetic and hormonal factors, an imbalance in food intake and energy expenditure, metabolic abnormalities, and the deregulation of autophagy [33,155].

Several comprehensive reviews have highlighted autophagy as a key factor underlying obesity [31,32,33]. As a catabolic mechanism, autophagy plays a pivotal role in maintaining physiological homeostasis by degrading and clearing obesity-related excesses, such as the accumulation of lipid droplets, protein aggregates, oxidative stress and damaged mitochondria [156,157]. Moreover, the removal of damaged mitochondria by mitophagy is crucial during protection against the development of insulin resistance and increased adiposity [158]. Hence, pathological alterations in autophagy should certainly exacerbate disorders associated with obesity.

Recent studies have associated the function and regulation of autophagy with the low-grade inflammation characteristic of obesity. The inflammation type exhibited by obesity is unique, affecting multiple organs (e.g., the liver, heart and pancreas) as well as skeletal muscle and adipose tissue [159]. In addition, inflammation has a considerable impact on metabolic homeostasis. 

Since macrophages are the most important cells participating in obesity-related inflammation, they have been the focus of research on the immune response in adipose tissue under conditions of obesity [160]. The homeostasis of adipose tissue implies the coordinated activity of resident immune cells. The particular phenotype of resident macrophages is determined by the pattern of secretion of cytokines by T lymphocytes. A normal diet evokes the predominant expression of M2 (alternatively activated) macrophages, while a high-fat diet gives rise to a switch from the M2 polarization phenotype to a proinflammatory M1 polarization state in macrophages of adipose tissue. The latter phenotype contributes to an over-production of proinflammatory cytokines (e.g., TNF-⍺, IL-6 and IL-12) and ROS [161]. Thus, the change from the M2 to M1 phenotype implies a conversion of the macrophage function from protective to damaging. Furthermore, the impaired autophagy that occurs in macrophages with obesity significantly upregulates the production of ROS, inducing systemic insulin resistance and exacerbating atherosclerosis [162,163]. Thus, impaired autophagy in macrophages perpetuates and promotes proinflammatory M1 polarization, highlighting the critical role of autophagy in macrophage polarization and obesity [164]. 

Many efforts have been made to explain the association between comorbidities and the severity of COVID-19. According to one proposal, obesity leads to a larger vulnerability to infections because adipose tissue serves as a reservoir for several viruses, including HIV, cytomegalovirus, and SARS-CoV-2. In some cases, obesity fosters an exceptional form of viral pathogenesis involving the destruction of adipocytes and other tissues by memory T cells [151,165]. Some antihypertensive drugs are related to increased ACE2 expression, which is utilized by SARS-CoV-2 to enter cells [42]. Moreover, the positive modulation of IL-6R and IL-6 expression in the adipose tissue of obese individuals may participate in the uncontrolled inflammation observed in COVID-19 [166]. Based on the structural and proteomic similarities between SARS-CoV and SARS-CoV-2, the latter was recently suggested to affect host metabolism as part of its lifecycle [167]. This theory emphasizes the possible ability of SARS-CoV-2 to deregulate lipid autophagy (lipophagy) in favor of its viral lifecycle. It is then likely that autophagy contributes to the close correlation between obesity and the severe clinical manifestations of COVID-19.

## 6. Pharmacological Intervention Targeting Autophagy

Although further research is needed on the intricate relation between viral infections and autophagy, the information encountered in the literature justifies the plausible therapeutic efficacy of inhibiting or activating this cellular mechanism. The current focus is on a limited number of inhibitors and activators of autophagy, given the strategy of targeting the antiviral response to diminish viral replication in the first phase of COVID-19, and the exaggerated inflammation to avoid complications in the third phase of the disease (Table 2). It is important to consider the wide range of autophagic activity to avoid negative consequences. Numerous compounds are being investigated nowadays as up- or downregulators of autophagy. Some authors have provided a comprehensive summary of autophagy-related drugs or compounds as novel treatments against SARS-CoV-2 [168,169].

There are several autophagy inhibitors with promise for a positive intervention in the pathogenesis of anti-SARS-CoV-2. For instance, the first drugs suggested for the treatment of COVID-19 were chloroquine (CQ) and its less toxic derivative, hydroxychloroquine (HCQ) [170], which are known to inhibit autophagic flux by interfering with autophagosome–lysosome fusion, increasing the endosomal/lysosomal pH. Due the broad-spectrum antiviral effects of CQ and HCQ, their applications have been proposed against HIV, SARS-CoV and Zika [171,172]. In vitro, HCQ is effective for inhibiting the entry step, as well as the post-entry stages of SARS-CoV-2, in Vero E6 cells. This effect is due to changing the glycosylation of ACE2 receptor and spike protein, in addition to the failure of the transport of virions to the releasing site by blocking endosomal maturation [173]. Some limited clinical trials were conducted in cohorts of patients with COVID-19 which have suggested the efficacy of CQ and HCQ to mitigate SARS-CoV-2-induced pneumonia and decrease the mortality rate [174,175]. Contrarily, other clinical studies do not find clinical benefits of HCQ for COVID-19 [176,177]. The discrepancy between studies probably reflects differences in patients enrolled, design of the studies, dosages and timeline of pathogenesis of COVID-19. This asseveration is supported by some studies which have showed some benefits of HCQ in early and mild COVID-19, mainly associated with viral load reduction [178,179].

**Figure 4:**
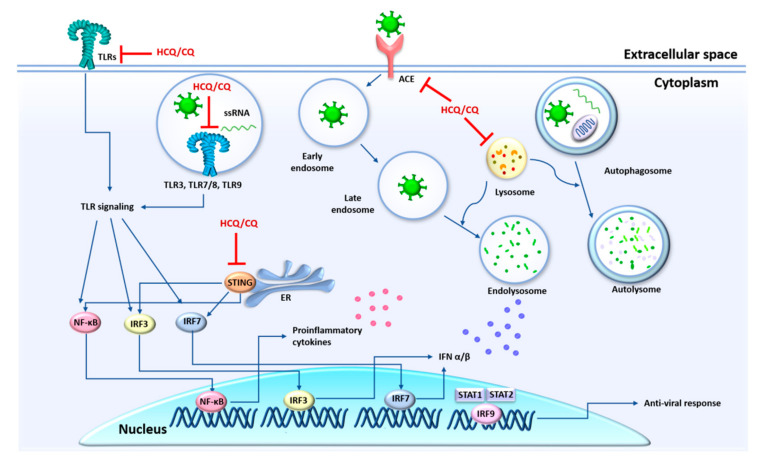
Main action mechanisms of chloroquine (CQ) and hydroxychloroquine (HCQ) related to antiviral and immunomodulators properties. By increasing the pH into lysosomes, CQ and HCQ interfere with cargo derived from endocytosis and/or from autophagic pathway. Moreover, CQ and HCQ prevent the PAMPs’ recognition by Toll-like receptors (TLRs). These drugs can inhibit the TLR and STING signaling, which automatically decreases the production of pro-inflammatory cytokines including the IFN response.

HCQ and CQ can inhibit certain cellular functions and molecular pathways involved in immune activation. For instance, HCQ and CQ interfere with TLR [180] and STING [181] signaling, which prevents the production of pro-inflammatory cytokines, including IFN response. The various modes of action of these drugs make their use in therapeutic interventions for COVID-19 difficult. It is likely that their applications are dependent on the inflammatory conditions of the disease [182]. Although severe adverse effects led to the suspension of clinical trials and their therapeutic use to treat COVID-19 [183], more in vitro studies and clinical trials are needed to understand the direct and indirect action mechanisms of these drugs to improve the CQ and HCQ-based COVID-19 treatments (Figure 4).

Lysosomotropic compounds ARN5187 and Lys05 are also good candidates for a clinical trial to evaluate their antiviral activity against SARS-CoV-2. They block the final maturation of autolysosomes and thus inhibit autophagy [184,185]. Eugenol and evodiamine are other autophagy inhibitors with promise for treating COVID-19, given the evidence of their antiviral properties against the influenza A virus. Whereas the former interferes with autophagy by avoiding the dissociation of Beclin1-Bcl2, the latter inhibits the formation of the Atg5-Atg12/Atg16 heterotrimer [186,187]. 

Other plausible therapeutic alternatives are inhibitors of the ULK complex or PI3-K in order to disable autophagy. Accordingly, inhibitors of the ULK complex that suppress autophagy and the autophagic flux are ULK-100, ULK-101 [188], compound 6 [189], MRT67307, MRT68921 [190] and SBI-0206965 [191]. Among the inhibitors of PI3K commonly employed to block autophagy are 3-methyladenine [192], wortmannin, LY294002 [193], PT210 [194] and GSK-2126458 [195]. Inhibitors of VPS34 (a PI3K) are VPS34-IN1 and VVPS34-IN1 [103,104], which have antiviral properties against SARS-CoV-2. The analogues of these inhibitors should also be investigated: Spautin-1 [196], SAR405 [197], compound 31 [198] and PIK-III [199].

In the third and critical stage of COVID-19 (characterized by an uncontrolled inflammatory response), it may be advantageous to administer activators of autophagy. Some drugs approved by the FDA for the treatment of other diseases act as autophagy inducers, although their mechanisms and their capacity for promoting autophagy are not yet clear. For instance, metformin is an oral anti-diabetic drug frequently prescribed to suppress glucose production in the liver. It has also been assessed for its anticancer effect. A recent review has mentioned the positive impact of metformin on the prognosis of hospitalized patients with diabetes type 2 and COVID-19 [200]. The author did not point to autophagy as the means of restoring homeostasis, but this drug is a known promotor of autophagy through the activation of AMPK and regulation of mTOR [201], mechanisms related to its anti-inflammatory effect [202]. 

Vitamin D3, a pleiotropic hormone with activity against intracellular *Mycobacterium tuberculosis*, has the capacity to evoke autophagy [203]. Some studies have found that vitamin D3 supplementation reversed a strong inflammatory response [204]. However, it must be studied more rigorously in relation to the activation of autophagy before considering randomized clinical trials on its efficacy for treating COVID-19. Spermidine, a natural polyamine, has been correlated with the control of cytomegalovirus infection. It restores homeostasis in deregulated autophagy and reestablishes CD8 (+) T cell memory formation by an autophagy-dependent process capable of improving immunity [205,206]. Resveratrol has well-recognized antiviral and anti-inflammatory activity, and the latter seems to be related to autophagy in endothelial cells [207]. The therapeutic potential of resveratrol against emerging respiratory viruses has been discussed [208]. For cytomegalovirus infection, trehalose (an activator of autophagy) has been suggested as a therapeutic antiviral approach [209] and proposed as a potential preventative treatment for SARS-CoV-2 infection [210].

Additionally, there are other drugs with potential antiviral efficiency against SARS-CoV-2 in evaluation. Nitazoxanide is a commercial antiprotozoal agent with antiviral potential against a broad range of viruses including MERS-CoV and other coronaviruses [211]. Nitazoxanide induces autophagy and inhibits the intracellular proliferation of *M. tuberculosis* [212]. Ivermectin, a drug known as a specific inhibitor of nuclear import mediated by Importin alpha/beta and an in vitro inhibitor of SARS-CoV-2 replication [5], has been reported as an inductor of autophagy through the AKT/mTOR signaling pathway [213]. Emtricitabine and Tenofovir have shown moderate reduction of the overall clinical scores of SARS-CoV-2 infected ferrets [214]; these drugs modulate autophagy through the increased expression and accumulation of SQSTM1/p62 [215] and block the autolysosomes’ formation [216], respectively. For these drugs, several action modes have been described, and further in vitro studies and clinical trials are needed to establish if their antiviral properties are due to their influence over autophagy.

In addition to pharmacological agents to modulate autophagy, the use of light against COVID-19 is in an exploratory stage, predominantly photothermal and ablation techniques applied in critical cases to avoid catastrophic effects [217]. These ultrashort laser pulses may provoke sharp and selective photodamage at specific subcellular sites and thus trigger signaling pathways. Photo-induced regulation of transcription factors eventually gives rise to autophagy [218]. The production of ROS resulting from photothermal activity was correlated with the downregulation of the Akt-mTOR-p70S6K pathway, which turns on autophagy [219]. Although laser therapy for coronavirus is still in its infancy, it represents an opportunity to precisely stimulate cell processes involved in the regulation of the immune response (e.g., autophagy). Theoretical calculations and practical experimentation alike will be required to develop this technology.

Despite the promising therapeutic strategy of targeting autophagy to treat COVID-19, especially in the first and the third stages of the disease, further research is mandatory to define the advantages and disadvantages in regard to efficacy and safety. The ectopic modulation of autophagy represents an approach of remarkable clinical significance. Drugs targeting autophagy may be employed to counteract the evolutionary strategies developed by several viruses to harness the autophagy machinery in their replication and propagation process. Moreover, the modulation of autophagy also provides a potential treatment in the current condition for COVID-19, where there are no antiviral specific drugs. Since autophagy is a central modulator of innate and adaptative immunity, its modulation can provide an effective solution avoiding the exacerbated immune response and improving the morbidity and mortality rates in patients with severe COVID-19. Moreover, the autophagy-activated drugs offer the ability to restore homeostasis in diseases associated with dysfunctional autophagy to balance the antiviral and inflammatory response. In either situation, the decision to inhibit or activate the autophagy seems to depend on various factors, such as age, nutritional health conditions, genetic and the pre-existence of diseases associated with autophagy defects. Furthermore, since autophagy has a fundamental role in a plethora of signaling pathways, future studies seem to be essential to clarify its impact on the activation/deactivation of several cell mechanisms.

## 7. Conclusions

**Figure 5:**
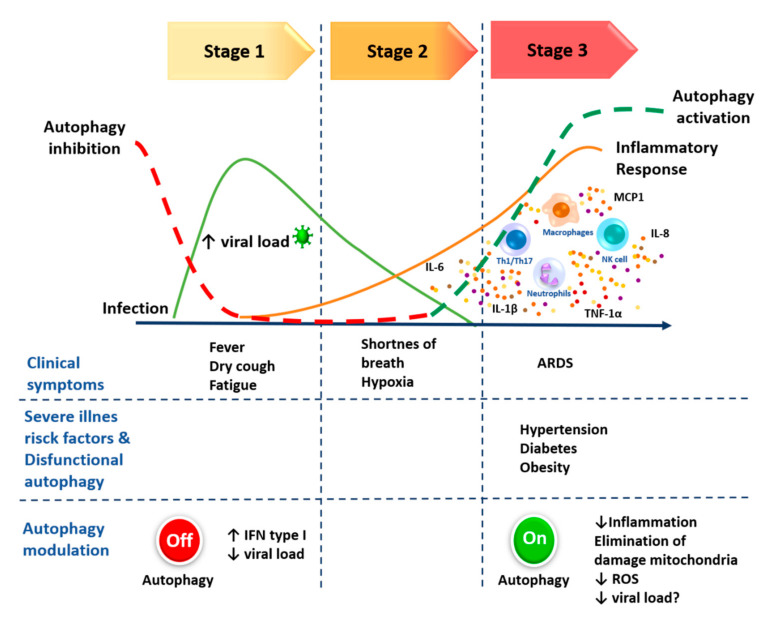
Schematic proposal to modulate autophagy in the context of the pathological timeline of COVID-19. Pharmacological inhibition of the autophagy at the first stage might regulate the IFN antiviral responses and restrict viral replication. Activation of autophagy at the third stage could contribute to the elimination of reactive molecules as ROS, to the removal of damaged organelles to decrease the inflammation and to restoring the balance of the immune response.

The complexity of the cellular and multiorgan disorders caused by SARS-CoV-2 requires novel and sophisticated therapeutic approaches. One possibility is to target autophagy, which could be responsible for the ineffective immune response that is unable to control viral replication and therefore ends up promoting spiraling inflammation. In light of the literature considered here, it is plausible to propose the inhibition of autophagy in the first stage of COVID-19 to prevent SARS-CoV-2 from taking advantage of this process to replicate itself. Moreover, since autophagy acts as a negative regulator of IFN response, its inhibition might restore the antiviral efficiency of the immune response to control the viral replication. On the other hand, uncontrolled inflammation is the hallmark of COVID-19 in its third and most severe stage. As it has been highlighted in the current review, autophagy participates in diverse signaling pathways in the antiviral immune response, including many involved in inflammation. Moreover, the comorbidities associated with severe COVID-19 have been related to dysfunctional autophagy. Therefore, activation of autophagy in this critical stage represents a potential approach to regulate the exacerbated immune response and restore homeostasis (Figure 5). Thus, the modulation of autophagy to restore homeostasis in the immune response to COVID-19 represents an important challenge, implying the ability to improve the antiviral response, limit inflammation and avoid provoking other complications. To date, pharmaceutical intervention in the antiviral and anti-inflammatory pathways of autophagy during the pathogenesis of COVID-19 has not received adequate attention as a clinical target, in spite of the fact that it plays a fascinating and vital role in governing the viral mechanisms capable of sequestering the host immune processes. Even though targeting autophagy could be an efficient strategy for treating COVID-19, it is essential to acquire an in-depth understanding of the interplay between the pathological characteristics of the disease and the host mechanisms of autophagy that participate in the control of viral replication and regulation of the inflammatory response. Future studies are needed to improve knowledge of such mechanisms, as well as clinical trials to test drugs that target autophagy through mono- or combination therapy with autophagic inhibitors or activators of specific pathways. 

## Figures and Tables

**Table 1:** The main treatments that have been administered to COVID-19 patients.

Treatments	Mechanisms of Action	Type of Study	Main Results	Ref.
Remdesivir	A monophosphoramidate prodrug of an adenosine analogue that inhibits viral RNA polymerases	(a) Clinical trial (b) Compassionate use	(a) No association with statistically significant clinical benefits. (b) Clinical improvement in 36 of 53 patients (68%).	(a) [1] (b) [2]
Lopinavir/Ritonavir	A co-formulation of two structurally related protease-inhibitors as antiretroviral agents (HIV type 1 aspartate protease inhibitors)	Clinical trial	No significant benefit from the treatment compared to standard care.	[3]
Favipiravir plus IFN-α	Inhibits the RNA-dependent RNA polymerase (RdRp) of RNA viruses	Open label control study	Attenuated disease progression and improved viral clearance.	[4]
Ivermectin	A synthetic derivative of a macrocyclic lactone antiparasitic agent. Inhibits the nuclear import of host and viral proteins	In-vitro antiviral activity against SARS-CoV-2	Compared to the DMSO-treated control, a 93% reduction in viral RNA and a 99.9% in cell-associated viral RNA.	[5]
Hydroxychloroquine and Chloroquine	Both drugs accumulate in lysosomes, leading to elevated intra-vesicular pH that prevents endosome trafficking and viral fusion. They also interfere with the glycosylation of ACE-2 receptors, which prevents their binding by SARS-CoV-2 and thus infection.	(a) Prospective randomized trial (b) A pilot observational study (c) Discontinued by WHO	(a) No significant difference between patients with hydroxychloroquine + conventional treatment and those with the conventional treatment alone. (b) Clinical improvement in all participating patients receiving co-administration of hydroxychloroquine with azithromycin.	(a) [6] (b) [7] (c) [8]
Interferon (IFN)-α	A broad-spectrum antiviral agent	Clinical trials in process	IFN-β1a and IFN-α2b are currently being evaluated as potential candidates for the treatment of patients with COVID-19.	[9]
Arbidol/lopinavir/ritonavir	By inhibiting the virus-mediated fusion with the target membrane, arbidol blocks virus entry into the target cells	(a) Retrospective cohort study (b) Cohort of 50 patients in two groups: lopinavir/ritonavir regimen (34 cases) and arbidol alone (16 cases)	(a) A significant increase in the conversion rate from positive to negative results for the coronavirus test on days 7 and 14 for patients receiving arbidol plus lopinavir/ritonavir versus monotherapy with lopinavir/ritonavir. (b) After 14 days of treatment, there was no viral load for the arbidol-treated group, but a 44.1% viral load for the lopinavir/ritonavir-treated group.	(a) [10] (b) [11]
Tocilizumab	A humanized anti-interleukin-6-receptor (IL-6R) monoclonal antibody that inhibits IL-6	(a) Retrospective observational study (b) Cohort of 100 patients (c) Retrospective study	(a) No attenuation of the disease in critically ill patients after a single dose of tocilizumab. (b) A rapid and sustained positive response to tocilizumab treatment. (c) Alleviation of the clinical symptoms and avoidance of severe COVID-19 with tocilizumab treatments.	(a) [12] (b) [13] (c) [14]
Convalescent plasma therapy	Appears to exhibit a neutralizing antibody response directed against the viral S protein. The antibodies block SARS-CoV-ACE2 entry.	(a) Evaluation of 6 COVID-19 patients (b) Case series analysis of 5 critically ill patients (c) Open-label, multi-center, randomized clinical trial	(a) Effective in alleviating patient symptoms and ameliorating radiological injuries. (b) Improved clinical status of patients. (c) No statistically significant improvement in the clinical condition of patients.	(a) [15] (b) [16] (c) [17]
Corticosteroids	Anti-inflammatory effects are due to a negative regulatory mechanism (transrepression).	Cohort of 41 patients	Suppressed lung inflammation in 21% of patients.	[18]
Prezcobix	HIV protease inhibitor	Under clinical trials	The primary endpoints included symptom improvement and virus nucleic acid turning negative, but the optimal endpoint has not been determined.	[19]
Oseltamivir	Neuraminidase inhibitor.	(a) COVID-19 patients (75) (b) Non-severe and severe COVID-19 patients (393)	(a) Recovery rate: 31%; Mortality rate: 11%. (b) No significant improvement in the clinical condition of patients.	(a) [20] (b) [21]

**Table 2:** Some compounds that affect autophagy, with their respective mechanism of action.

Compounds	Effect on Autophagy	Mechanism of Action *	FDA Approval	Reference
Chloroquine (CQ) and Hydroxychloroquine (HCQ)	Inhibitors	Interfere with autophagosome-lysosome fusion.	Yes	[220]
ARN5187 Lys05	Inhibitors	Block autophagosome maturation.	No Yes	[184,185]
Eugenol	Inhibitor	A decline in oxidative stress and activation of ERK1/2, p38MAPK and IKK/NF-κB; downstream, lesser dissociation of the Beclin1-Bcl2 heterodimer and reduced autophagy.	No	[186]
Evodiamine	Inhibitor	A decreased formation of the Atg5-Atg12/Atg16 heterotrimer and expression of Atg5, Atg7 and Atg12.	No	[187]
Berberine derivatives	Inhibitors	Diminished activation of the MEK/ERK signaling pathway.	No	[221]
ULK-100 ULK-101 Compound 6 MRT67307 MRT68921 SBI-0206965	Inhibitors	Inhibition of the ULK complex.	No	[188,189,190,191]
3-methyladenine Wortmannin LY294002 PT210 GSK-2126458	Inhibitors	Inhibition of PI3K.	No	[192,193,194,195]
VPS34-IN1 VVPS34-IN1 Spautin-1 SAR405 Compound 31 PIK-III	Inhibitors	Inhibition of VPS34.	No	[103,104,196,197,198,199]
Spermidine	Activator	An increase in the expression of acetyltransferase EP300, known to bind to crucial autophagy proteins (Beclin1 and LC3) and stimulate autophagy.	Substance registration system	[206]
Nonsteroidal anti-inflammatory drugs (NSAIDs): Celecoxib Sodium Salicylate Aspirin Sulfasalazine Piroxicam Indomethacin	Activators	Modulation of autophagy through the signaling pathways of PI3K/Akt/mTOR, MAPK/ERK1/2, P53/DRAM, AMPK/mTOR, Bip/GRP78, CHOP/GADD153 and HGF/MET.	Yes	[222]
Rapamycin and derivative compounds (RAD001, CCI-779 and AP23573). AZD8055 Torin 1 Metformin	Activators	Inhibition of mTOR.	Yes No Yes	[223]
Vitamin D3	Activator	Activation of autophagy, though the pathway is unclear. Stimulation of calcium signaling is a proposed mechanism.	No	[224]
Resveratrol	Activator	Activation of autophagy by triggering the cAMPPRKA- AMPK-SI RT1 signaling pathway.	Clinical trial on animals (cancer therapy).	[225]
Trehalose	Activator	Activity independent of mTOR.	A food additive.	[209]

* Only mechanisms of action associated with autophagy are mentioned. Some compounds act on other cellular processes.
